# Comparison of Subgingival Irrigation Effect of Boric Acid 0.5% and Povidone-Iodine 0.1% on Chronic Periodontitis Treatment

**DOI:** 10.3290/j.ohpd.a45356

**Published:** 2020-10-02

**Authors:** Thuy Anh Vu Pham, Nhat Dinh Phan

**Affiliations:** a Associate Professor, Division of Odonto-Stomatology, School of Medicine, Vietnam National University, Ho Chi Minh City, Vietnam. Idea, analysed the data, wrote the manuscript.; b Specialist, Department of Periodontology, National Hospital of Odonto-Stomatology, Ho Chi Minh City, Vietnam. Collected and analysed the data, wrote the manuscript.

**Keywords:** boric acid, chronic periodontitis treatment, povidone-iodine, subgingival irrigation,

## Abstract

**Purpose:**

To comparatively evaluate the effect of a 5% boric acid (BA) irrigant on periodontal condition, bacterial level and oral neutrophil numbers with a 1% povidone iodine (PVP-I) irrigant as an adjunct to scaling and root planing (SRP) in chronic periodontitis (CP) treatment.

**Materials and Methods:**

A single-masked, randomised clinical trial with 36 CP patients was conducted at the Faculty of Odonto-Stomatology, University of Medicine and Pharmacy, Ho Chi Minh City, Vietnam. Subjects were randomly divided into two treatment groups: 1) SRP plus PVP-I 0.1% irrigant and 2) SRP plus BA 0.5% irrigant. Clinical measurements, including the plaque index (PI), gingival index (GI), bleeding on probing (BOP), probing depth (PD), clinical attachment level (CAL), bacterial level in subgingival plaque (BANA test) and the quantification of oral neutrophils were evaluated at baseline, 4, 6 and 8 weeks after treatment (T_0_, T_4_, T_6_ and T_8_).

**Results:**

Whole-mouth (PI, GI, BOP, PD, CAL and PD) parameters, bacterial level in subgingival plaque and number of oral neutrophils decreased statistically significantly after treatment compared to baseline in both groups (p < 0.01). Between the two groups, whole-mouth PI, GI, BOP, PD and CAL reduction in the BA 0.5% group were higher than those in the PVP-I 0.1% group, but statistical significance was found only for GI and BOP after treatment (p < 0.05). The PD and CAL reductions for moderately deep pockets (PD ≥ 5 mm and < 7 mm) were significantly greater in group 2 compared to group 1 after treatment compared to baseline (p < 0.01). This difference was not found for deep pockets (PD ≥ 7 mm).

**Conclusion:**

The results of this study suggest that BA 0.5% could be an alternative to PVP-I 0.1%, and might be more favourable because it provided superior results regarding whole-mouth BOP, GI as well as PD and CAL reduction for moderately deep pockets after CP treatment.

Chronic periodontitis (CP) is a chronic inflammatory disease that results in the destruction of connective tissues and structures surrounding the teeth. It manifests as periodontal pocket formation and/or clinical attachment loss, which is caused by certain bacteria existing in dental plaque.^2^ The gold standard procedure for periodontal treatment is mechanical therapy, consisting of scaling and root planing (SRP). However, due to limited access to the root surface and the tissue-invading properties of some periodontal pathogenic bacteria, mechanical therapy may be ineffective.^16,27^ Three bacteria, i.e. *Porphyromonas gingivalis* (Pg), *Treponema denticola* (Td) and *Tannerella forsythia* (Tf) are referred to as the “red complex”, as a large number of these bacteria are present in subgingival plaque and play important roles in the pathogenesis of CP.^15^ These bacteria can be detected using enzyme treatment on plaque samples to hydrolyse the synthetic peptic benzoid DL arginine-B-napthilamide (BANA). Often, supportive antibiotic or antimicrobial irrigation is necessary for periodontal pocket treatment. Subgingival irrigation with povidone iodine (PVP-I) and chlorhexidine (CHX) have shown limited success in CP treatment because of their potential toxicity and the complicated structure of the periodontal pocket.^14,24^ Povidone iodine is a commonly used antiseptic agent in medicine, but with some disadvantages; for instance, it can induce an allergic reaction, and should not be used in patients with thyroid function disorders or in pregnant and breastfeeding women.

**Fig 1:**
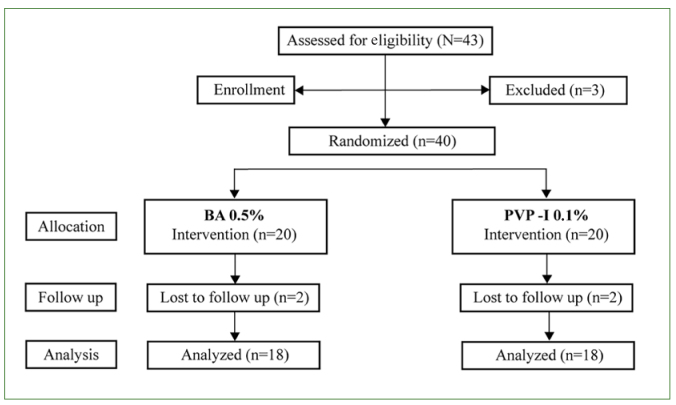
Study flowchart

Benkovic et al^5^ reported on the antibacterial activity of boron, which is a common element found in diverse foods, e.g. vegetables, fruits and nuts. Luan et al^23^ suggested that a compound containing boron, ANO128, has antibacterial and anti-inflammatory properties. ANO128 has been shown to reduce inflammation and bone loss in rats, and may be effective against some CP-related bacteria such as *Prevotella intermedia*, Pg, *Eubacterium nodatum* and Td. Sağlam et al^32^ showed that systemic administration of boric acid may reduce alveolar bone loss by affecting the receptor activator of nuclear factor kappa-B ligand (RANKL)/osteoprotegerin balance in periodontal disease in rats. Uysal et al^35^ have demonstrated that dietary boron (3 mg/kg daily, oral administration) has a positive effect on the early phase of bone regeneration of the mid-palatal suture in response to expansion and may be beneficial in routine maxillary expansion procedures with no side-effects in rabbits. In a recent study, Kanoriya et al^18^ demonstrated that boric acid (BA) 0.75% gel significantly decreased PD, CAL and the radiographic defect depth when combined with SRP for six months post-treatment. The reductions were statistically significantly higher than those seen with placebo.

Neutrophils are the most abundant type of white blood cell (50%-70%) and serve as a primary defense against infection. The typical response to infection or serious injury is increased neutrophil production.^4^ In healthy people, the leukocytes present in saliva – mainly from the gingival sulcus – are predominantly neutrophils.^19,20^ Neutrophils account for over 90% of cells in the gingival sulcus, providing a barrier between the conjunctive epithelium and subgingival biofilm to prevent apical deposition of bacterial biofilm.^4^ Neutrophil quantification to assess periodontal disease status and the efficacy of periodontal therapy was first proposed by Raeste and Aura.^29^ In that study, the authors concluded that the oral rinse assay for neutrophil counts provides information about the severity of inflammation. Previous studies have demonstrated that periodontal disease leads to an increased neutrophil count in the oral cavity. The hypothesis of this study was that BA 0.5% would have a better effect than povidone-iodine 0.1% for the treatment of CP. Thus, the aim of this study was to compare the effect of BA 0.5% on the periodontal condition, BANA bacterial level and oral neutrophil counts in comparison to the effect of PVP-I 0.1% irrigation as an adjunct to SRP in CP treatment.

## Materials and Methods

### Study Design and Patient Selection

This was a single-blind, randomised clinical trial with 36 CP patients ages 29 to 65 years, performed at the Department of Periodontology, Faculty of Odonto-Stomatology, University of Medicine and Pharmacy, Ho Chi Minh City, Vietnam. The ethics committee and review board of this University of Medicine and Pharmacy approved the research protocol (reference number: 500/DHYD-HD). Participation was voluntary, and written informed consent was obtained from all patients after ethical approval was granted. The inclusion criteria were: 1) patients diagnosed with moderate or severe CP according to the standards of the American Academy of Periodontology;^2^ patients had at least one gingival bleeding site, a periodontal pocket depth ≥ 5 mm or clinical attachment loss ≥ 3 mm and alveolar bone resorption in the radiographic image ≥ 16% or > 3 mm of root length; 2) patients with at least eight sites at which the periodontal pocket depth was ≥ 5 mm; 3) patients who had at least three teeth with a periodontal pocket ≥ 5 mm. The exclusion criteria were: 1) uncooperative patients, patients who did not follow instructions or withdrew from the study; 2) patients with aggressive periodontitis or periodontitis associated with endodontic lesions; 3) patients who had received periodontal treatment within the last 12 months; 4) patients with acute oral inflammation; 5) patients who had an allergic reaction to iodine and boric acid (alteration in sense of smell and/or taste, burning sensation, vomiting during gargling with PVP-I or BA); 6) patients who had used antibiotics or anti-inflammatory drugs within the last 6 months; 7) patients with general disorders and risk factors (such as diabetes, cardiovascular disease, HIV, smoking); 8) pregnant women or patients using hormonal products; 9) patients with haematological diseases or leukocyte disorders.

### Preparation of PVP-I 0.1% and AB 0.5%

PVP-I and AB (BASF; Ludwigshafen, Germany) were diluted into the concentrations of PVP-I 0.1% and AB 0.5% solutions by Department of Pharmaceutics, University of Medicine and Pharmacy, Ho Chi Minh City, Vietnam.

### Initial Visit

All patients were provided with information on the causes and consequences of periodontal disease and instructions on how to perform proper oral hygiene. Supragingival plaque retention factors were removed and cavities were filled.

### Periodontitis Treatment

At the second appointment, within 1 week after the initial visit, subjects had periodontitis treatment. They were divided into two groups: 1) SRP + PVP-I 0.1% irrigation, and 2) SRP + BA 0.5% irrigation. Patients who were diagnosed with CP were randomly allocated to one of the two treatment groups by an experienced investigator who did not collect data or perform periodontitis treatment. Periodontitis treatment including oral hygiene instruction, SRP along with proper root surface debridement using Gracey curettes (Hu-Friedy, Chicago, IL, USA) and irrigation (PVP-I 0.1%/group 1 or BA 0.5%/group 2) for all patients was performed with the same protocol by one periodontologist who was blinded to the type of antiseptic solution. Each periodontal pocket was irrigated with a volume of 10 ml PVP-I 0.1% or AB 0.5% for 10 s immediately after SRP. No antiplaque or antiseptic mouthwashes or antibiotics were prescribed after treatment. In each patient, SRP along with proper root surface debridement and irrigation was done and finished at the second visit.

### Collection of Oral Neutrophils

After obtaining written informed consent from all patients, supragingival calculus was removed and oral hygiene instruction given at the first visit. Examination and data collection at T_0_ were carried out at the second visit. At T_0_, patients were requested not to eat or drink anything 30 min before data collection and to attend the clinic between 9:00 AM and 11:00 AM to collect saliva. Patients rinsed with distilled water 5 min before saliva collection, then rinsed with 15 ml of 0.9% physiologic saline solution for 30 s and spit into plastic cups. The collected sample was poured into a sterilised 15-ml tube. All samples were analysed on the day of collection at the Histology and Embryology department, Faculty of Medicine, University of Medicine and Pharmacy, Ho Chi Minh City, Vietnam.

Neutrophils were counted and quantified based on the standard protocol using a hemocytometer, which is a modification of the protocol by Wright et al.^37^

### BANA Test

BANA tests were carried out for each patient at each appointment. The procedure involved collecting a subgingival plaque sample from the three deepest periodontal pockets of three different teeth with a curette at the deepest point after evaluating the above-mentioned indices. Collection areas were air dried and isolated using sterile cotton wool. This evaluation was performed at T_0_, T_4_, T_6_ and T_8_. After collection, the curette was wiped in order to avoid cross-infection between samples. After sample collection, the subgingival plaque was placed on a prepared strip soaked with BANA (N-benzoyl-DL-arginine-2-napthylamide-zyme). Then, the samples were moisturised on the strip using a wet cotton swab. The strip was bent to ensure that all of the plaque sample was attached to the matrix surface. The sample was then incubated at 35°C for 5 min in an incubator dedicated to the BANA assay. The sample was assessed according to colour intensity: + 0 or negative, no colour; + 1 or weakly positive: pale blue pinpoints or diffuse patches; + 2 or positive: darker blue pinpoints or diffuse patches.^21^ The researcher performing this assay was trained and tested before participating in the analysis.

The following parameters were evaluated: clinical parameters including plaque index (PI), gingival index (GI), bleeding on probing (BOP), pocket depth (PD), clinical attachment loss (CAL); the bacterial level in subgingival plaque (BANA test) and oral neutrophil counts were evaluated at four time points: T_0_, T_4_, T_6_ and T_8_. PI and GI were examined according to Loe’s criteria.^22^ PD and CAL were measured using a UNC 15 periodontal probe (Hu-Friedy; Chicago, IL, USA). BOP was assessed as the presence or absence of bleeding 30 s after probing.^36^ All clinical examinations performed at baseline were repeated 4, 6, and 8 weeks after treatment by one calibrated dentist who was masked for allocation to the PVP-I 0.1% group or BA 0.5% group and did not perform the periodontal treatments in this study.

### Statistical Analysis

The impact of the treatment strategy on changes in PI, BOP, GI, PD, CAL, BANA test score and oral neutrophil counts (for baseline vs follow-up visit) was examined by repeated measures ANOVA combined with the Greenhouse-Geisser correction. Comparisons between two groups at the same time point were performed using the Tukey or Mann-Whitney test. A p-value < 0.05 was considered statistically significant.

## Results

Of the 40 patients initially enrolled in the trial, 36 completed the eight-week follow-up period. Two subjects in the PVP-I 0.1% group and two in the BA 0.5% group withdrew from the trial for personal reasons. There were 18 patients in the PVP-I group (11 males and 7 females, with a mean age of 51.1 ± 11.2 years, range 30 to 65 years) and 18 patients in the BA group (7 males and 11 females, with a mean age of 48.8 ± 12.2 years, range 29 to 69 years). No statistically significant differences in the demographic and clinical variables were found between the two treatment groups at baseline.

### Whole-Mouth PI, GI, BOP, PD and CAL

The results of the whole-mouth clinical measurements (mean ± SD) between baseline and all time points in the treatment groups are displayed in Tables 1 and 2. At T_4_, T_6_ and T_8_, all patients showed a statistically significant improvement in oral hygiene compared with baseline in all groups (p < 0.05).

At baseline, no statistically significant differences in clinical parameters were found among treatment groups. At all points time after treatment, no statistically significant differences in PI, PD or the CAL index were found between the two groups. However, the BA 0.5% group showed statistically significantly greater GI and BOP percentage reduction at 4, 6 and 8 weeks than did the PVP-I 0.1% group (p < 0.05) (Table 1).

**Table 1:** Whole-mouth clinical parameters of two groups at four time points (mean ± SD)

Parameters	T_0_	T_4_	p*	T_6_	p*	T_8_	p*
PI							
PVP-I 0.1%	1.11±0.32	0.76±0.25	< 0.001	0.68±0.21	< 0.001	0.66±0.21	< 0.001
BA 0.5%	1.10±0.44	0.68±0.33	0.001	0.67±0.32	0.001	0.66±0.31	0.001
p**	0.98	0.39		0.92		0.99	
GI							
PVP-I 0.1%	1.42±0.33	1.05±0.37	< 0.001	1.00±0.36	< 0.001	0.95±0.34	< 0.001
BA 0.5%	1.39±0.36	0.76±0.40	< 0.001	0.74±0.39	< 0.001	0.66±0.40	< 0.001
p**	0.8	0.03		0.046		0.043	
BOP (%)							
PVP-I 0.1%	46.15±14.9	25.33±12.30	< 0.001	24.25±10.31	< 0.001	23.30±10.60	< 0.001
BA 0.5%	51.51±14.75	13.19±6.57	< 0.001	12.63±6.45	< 0.001	12.07±5.89	< 0.001
p**	0.42	0.001		< 0.001		< 0.001	

Different letters mean statistically significant differences among groups. * The p-value was calculated by repeated measures ANOVA combined with the Greenhouse-Geisser correction. ** The p-value refers to statistically significant differences between groups in the same period; Tukey’s test. A significance level of 5% was used.

**Table 2:** Differences in PD and CAL parameters (mean ± SD)

Parameters	T_0_	T_4_	p*	T_6_	p*	T_8_	p*
Whole mouth							
PD (mm)							
PVP-I 0.1%	3.04±0.73	2.52±0.59	< 0.001	2.46±0.77	< 0.001	2.43±0.84	< 0.001
BA 0.5%	2.93±0.43	2.35±0.47	< 0.001	2.34±0.79	< 0.001	2.30±0.80	< 0.001
p**	0.89	0.34		0.42		0.41	
CAL (mm)							
PVP-I 0.1%	3.85±1.00	3.36±0.81	< 0.001	3.28±0.51	< 0.001	3.24±0.52	< 0.001
BA 0.5%	3.47±0.88	2.95±0.81	< 0.001	2.87±0.47	< 0.001	2.84±0.45	< 0.001
p**	0.17	0.14		0.12		0.2	
Moderately deep pockets (5 mm≤PD<7 mm)							
PD (mm)							
PVP-I 0.1%	5.23±0.42	3.73±0.87	< 0.001	3.63±0.86	< 0.001	3.62±0.87	< 0.001
BA 0.5%	5.21±0.41	3.52±0.84	< 0.001	3.42±0.84	< 0.001	3.40±0.84	< 0.001
p**	0.51	0.005		0.01		0.02	
CAL (mm)							
PVP-I 0.1%	5.78±1.22	4.29±1.46	< 0.001	4.24±1.46	< 0.001	4.22±1.46	< 0.001
BA 0.5%	5.77±1.41	4.22±1.37	< 0.001	4.17±1.37	< 0.001	4.15±1.37	< 0.001
p**	0.5	0.002		0.04		0.045	
Deep pockets (PD≥7 mm)							
PD (mm)							
PVP-I 0.1%	7.78±0.79	6.02±1.09	< 0.001	6.00±1.09	< 0.001	5.99±1.07	< 0.001
BA 0.5%	7.76±0.96	5.93±1.08	< 0.001	5.90±1.08	< 0.001	5.89±1.06	< 0.001
p**	0.44	0.36		0.3		0.3	
CAL (mm)							
PVP-I 0.1%	8.64±1.36	6.94±1.37	< 0.001	6.90±1.37	< 0.001	6.89±1.35	< 0.001
BA 0.5%	8.38±1.42	6.63±1.47	< 0.001	6.60±1.47	< 0.001	6.59±1.45	< 0.001
p**	0.18	0.1		0.08		0.08	

Different letters mean statistically significant differences among groups. * The p-value was calculated by repeated measures ANOVA combined with the Greenhouse-Geisser correction. ** The p-value refers to statistically significant differences between groups in the same period; Tukey’s test. A significance level of 5% was used.

### Analysis of Moderately Deep Pockets (PD ≥5 mm and PD <7 mm)

The comparison of PD and CAL in moderately deep pockets before and after treatment also revealed a significant difference (p < 0.05) (Table 2). A total of 629 pockets were examined (PVP-I = 325; BA = 304). The PD and CAL reductions in moderately deep pockets were greater in the BA group compared with the PVP-I group at all time points after treatment (p < 0.05).

### Analysis of Deep Pockets (PD ≥7 mm)

The results of PD and CAL values for deep pockets are presented in Table 2. A total of 217 pockets were treated (PVP-I = 126; BA = 91). The PD and CAL values of both groups were not statistically significantly different at baseline (p > 0.05). Statistically significant reductions were found in PD and CAL for deep pockets in both groups at all time points after treatment compared with baseline (p < 0.05). Although no statistical significance was found, PD and CAL reductions in deep pockets in the BA 0.5% group tended to better greater than in PVP-I 0.1% group at all time points after the treatment.

### BANA Test

The bacterial level was determined according to the BANA test performed on three samples collected from the three deepest pockets of each patients. The results are presented in Table 3.

**Table 3:** Bacterial level determined using the BANA test score and oral neutrophil counts (x 10^6^ per ml saliva) at four time points (mean ± SD)

Variables	T_0_	T_4_	T_6_	T_8_	p*
BANA test					
PVP-I 0.1%	1.67±0.48	0.81±0.48	0.63±0.49	0.39±0.49	< 0.001
BA 0.5%	1.70±0.46	0.70±0.46	0.57±0.50	0.35±0.48	< 0.001
p**	0.09	0.09	0.1	0.09	
Oral neutrophil counts					
PVP-I 0.1%	3.71±1.04	2.52±0.86	2.41±0.81	2.37±0.77	< 0.001
BA 0.5%	3.63±1.71	2.30±1.29	2.18±1.23	2.01±1.17	< 0.001
p**	0.87	0.55	0.49	0.37	

*The p-value was calculated by repeated measures analysis of variance (ANOVA) combined with the Greenhouse-Geisser correction. **The p-value refers to statistically significant differences between groups in the same period; Tukey’s test. A significance level of 5% was used.

At baseline, no statistically significant differences among treatment groups were found. The bacterial level parameters were markedly reduced in both groups after treatment compared with baseline (p < 0.05). Although no statistically significant differences were detected, the BA 0.5% group showed better bacterial level reduction at all time points after treatment compared to the PVP-I 0.1% group.

### Quantification of Oral Neutrophil Counts

Oral neutrophil counts at all time points are presented in Table 3. At baseline, no statistically significant differences among treatment groups were found. Oral neutrophil counts were markedly reduced in both groups after treatment compared with baseline (p < 0.05). Although no statistically significant differences were detected, the BA 0.5% group tended to show greater reduction in the oral neutrophil counts at all time points after treatment compared to the PVP-I 0.1% group.

## Discussion

This study demonstrated the efficacy of PVP-I 0.1% and BA 0.5% irrigation for reducing PI, GI, BOP, PD, the CAL index, the bacterial level and the oral neutrophil count after eight weeks of treatment. The BA group showed greater reductions in GI and BOP in the whole mouth as well as in PD and CAL in moderately deep pockets compared to the PVP-I group. Although no statistically significant difference were found, PD and CAL reductions in deep pockets, reduced BANA bacterial level, and oral neutrophil reduction in the BA 0.5% group tended to be better than in the PVP-I 0.1% group at all time points after the treatment.

PVP-I irrigation is commonly used and has been shown to be an effective periodontitis treatment.^33^ In this study, PVP-I was used as the control treatment in comparison with BA 0.5%. Many studies have shown that the dilution of PVP-I 10% increases it antibacterial action. Berkelman et al^6^ showed that a 100-fold dilution of PVP-I 10%, i.e. PVP-I 0.1%, has stronger and faster antibacterial action. This is due to the increase in free iodine following dilution. Caufield et al^7^ demonstrated that 0.1% to 0.5% iodine-containing solution had bactericidal effects on periodontal pathogens such as *Actinobacillus actinomycetemcomitans* (Aa), Pg and *Prevotella intermedia* upon subgingival irrigation. In a longitudinal study, Rosling et al^30^ demonstrated that PVP-I 0.1% led to a statistically significant decrease in periodontal PD and improved clinical attachment after 12 months of treatment. A follow-up period of 13 years after CP treatment in the PVP-I 0.1% group showed a statistically significantly lower CAL than in the physiological saline group.^30^

In this study, we evaluated the efficacy of BA irrigation for CP treatment in comparison to PVP-I irrigation regarding clinical and bacterial level parameters as well as oral neutrophil counts before and after eight weeks of treatment. No local or systemic allergies were recorded. In an in vitro/clinical study, BA 0.75% was found to be non-toxic to human gingival and periodontal fibroblasts.^31^ In a recent study by Kanoriya et al,^18^ a reduction in PD and a gain in clinical attachment at one month and three months after treatment as well as a decrease in bone resorption observed in radiographic images after six months of treatment with a 0.75% BA gel subgingivally after SRP were markedly higher than those observed with the placebo.^18^

Using an immunofluorescence method in this study, we evaluated oral neutrophil counts in patient saliva before and after 4, 6, and 8 weeks of treatment. Before conducting the study, we tested this method on four CP patients without any periodontal treatment and four healthy subjects. The saliva was collected twice, with the second collection conducted one week after the first. There was no statistically significant difference in oral neutrophil counts in samples taken at the two time points in each group.

Oral neutrophil counts of CP patients in both groups were markedly reduced after treatment, and a statistically significant decrease was also obtained in clinical periodontal parameters after treatment. These findings show that neutrophil counts in saliva can reflect the periodontal status as well as changes in condition before and after treatment. Although the BA 0.5% group showed greater reductions in neutrophil counts than the PVP-I 0.1% group, there was no statistically significant difference despite a decrease in whole-mouth BOP and GI as well as PD and CAL in moderately deep pockets in the BA group, with better effects of BA compared to PVP-I. This may be due to the small sample and short follow-up period. In future studies, a longer follow-up period is needed. Salivary neutrophil quantification using immunofluorescence is simple, rapid and very convenient. Changes in saliva contents are believed to reflect local and systemic diseases. Many studies have supported a relationship between neutrophil counts in saliva and CP condition. The results of this study agree with those of Bender et al.^4^ When comparing neutrophil counts in saliva between the healthy group and the CP group before and after treatment, Bender et al found a stable neutrophil number in the saliva of the healthy group. Furthermore, this number was statistically significantly lower than that of the CP group.^4^ The main function of neutrophils is to phagocytise and kill bacteria. More severe periodontitis is associated with a higher neutrophil number in response to increased bacterial numbers and desquamated epithelium at the bottom of periodontal pockets. The quantification of neutrophils in saliva can be a very useful tool for screening, detecting and monitoring the cause and prognosis of periodontal disease before and after treatment.^19^

In this study, PVP-I 0.1% yielded a poorer outcome than did BA 0.5%. Marsh and Bradshaw^25^ suggested that iodine has poor biofilm penetration ability. The efficacy of an antimicrobial system depends on antimicrobial concentrations being maintained for a sufficient period of time. However, according to Engstrom,^13^ the short duration of action of PVP-I is one reason it is not as efficaceous.^13^ Moreover, iodine can be immediately inactivated by organic substances in the deep periodontal pocket and is negatively affected by a number of biological factors, such as dentin complexes or collagen fibers.^26^

Ince et al^17^ reported that BA prevents oxidative damage by increasing levels of the antioxidant glutathione and increasing neutralisers of the oxidative reaction.^17^ Furthermore, there is a strong correlation between gingival crevicular fluid substances, peroxide concentrations and oxygen status with pocket depth or CAL.^1^ This may explain why pocket depth and CAL were markedly reduced in moderately deep pockets in the BA irrigation group.^31^ However, in deep pockets, due to the existence of a large amount of bacteria and the complexity of the anatomical structure, SRP combined with BA irrigation was not as effective.

It has been suggested that boron plays an important role in inflammatory and immune responses. Travers et al^34^ reported a statistically significant improvement in joint swelling and restricted movement upon boron supplement in rheumatoid arthritis patients. In this study, gingival bleeding was reduced in the BA group to a greater extent than in the PVP-I group, possibly as a result of the anti-inflammatory activity of boron.^34^ Additionally, it has been suggested that boron-containing AN0128 is effective against several periodontal pathogens such as *Prevotella intermedia*, Pg, *Eubacterium* *nodatum* and Td with a minimum inhibitory concentration less than 0.5 mg/ml.^23^ In the present study, the effect of PVP-I 0.1% in reducing pocket depth and CAL in moderately deep pockets poorer than that of BA 0.5%. Moreover, the decrease in gingival bleeding and gingival indices in the whole mouth after treatment demonstrated that BA was more effective in the early post-treatment period compared to PVP-I. This finding is consistent with the results of Sağlam et al^20^ when comparing BA vs CHX one month after treatment. In our study, the results show that both BA 0.5% and PVP-I 0.1% reduced Pg, Tf and Td numbers in subgingival plaque compared with baseline. This corresponded to the reduction in inflammation and PD in both groups post-treatment. Although the BA 0.5% group showed a greater reduction in bacterial levels at all time points after treatment, the differences were not statistically significant. This may be due to the small sample size of the study and the insufficient follow-up period.

Although the follow-up period of eight weeks in this study was relatively short, the results show that BA 0.5% provided promising outcomes for CP treatment as an irrigant combined with SRP because of the improved clinical healing parameters after treatment. Christgau et al^9,10^ showed statistically significant improvements of the clinical healing parameters (BOP, PPD, CAL) and statistically significant reductions of four of the investigated periodontal pathogens (Aa, Tf, Pg and Td) four weeks after SRP with hand instruments. The greatest change in PD and CAL reduction occurs within 1–3 months after SRP,^3,8^ although healing and maturation of the periodontium may occur over the following 9–12 months.^11,28^ Dahlén et al^12^ suggested that the response of the periodontium to SRP should be evaluated 4 weeks post-treatment. Additionally, this study suggests that salivary neutrophil quantification should be used for screening, early diagnosis and follow-up of periodontal disease. In the future, further in vitro studies and clinical trials are needed to assess BA at various concentrations for application in periodontal treatment in particular and oral diseases in general.

## Conclusion

BA 0.5% as a subgingival irrigant was found to have more beneficial effects as a CP treatment than PVP-I 0.1%, particularly in reducing whole-mouth BOP and GI as well as PD and CAL in moderately deep pockets. Although no statistically significant differences were found, PD and CAL reductions in deep pockets, BANA bacterial level and oral neutrophil counts reductions with BA 0.5% tended to be better than that with PVP-I 0.1% at all post-treatment time points. Future studies with a longer follow-up period and larger population are needed to fully elucidate the utility of boric acid in the treatment of destructive periodontal diseases.
